# Pan-Cancer Analysis Identifies CHD5 as a Potential Biomarker for Glioma

**DOI:** 10.3390/ijms23158489

**Published:** 2022-07-30

**Authors:** Lei Xu, Fengling Shao, Tengling Luo, Qijun Li, Dongmei Tan, Yi Tan

**Affiliations:** 1 Laboratory Animal Center, Chongqing Medical University, Chongqing 400016, China; xulei191052@cqmu.edu.cn (L.X.); luotl1128@126.com (T.L.); liqijun9889@163.com (Q.L.); dongmeitan@cqmu.edu.cn (D.T.); 2 The Ministry of Education Key Laboratory of Laboratory Medical Diagnostics, The College of Laboratory Medicine, Chongqing Medical University, Chongqing 400016, China; 2021130084@stu.cqmu.edu.cn

**Keywords:** CHD5, pan-cancer, immune microenvironment, glioma, biomarker

## Abstract

The chromodomain helicase DNA binding domain 5 (CHD5) is required for neural development and plays an important role in the regulation of gene expression. Although CHD5 exerts a broad tumor suppressor effect in many tumor types, its specific functions regarding its expression levels, and impact on immune cell infiltration, proliferation and migration in glioma remain unclear. Here, we evaluated the role of CHD5 in tumor immunity in a pan-cancer multi-database using the R language. The Cancer Genome Atlas (TCGA), Genotype Tissue Expression (GTEx), and Cancer Cell Lines Encyclopedia (CCLE) datasets were utilized to determine the role of CHD5 in 33 types of cancers, including the expression level, prognosis, tumor progression, and immune microenvironment. Furthermore, we explored the effect of CHD5 on glioma proliferation and migration using the cell counting kit 8 (CCK-8) assay, transwell assays and western blot analysis. The findings from our pan-cancer analysis showed that CHD5 was differentially expressed in the tumor tissues as compared to the normal tissues. Survival analysis showed that CHD5 was generally associated with the prognosis of glioblastoma (GBM), low Grade Glioma (LGG) and neuroblastoma, where the low expression of CHD5 was associated with a worse prognosis in glioma patients. Then, we confirmed that the expression level of CHD5 was associated with tumor immune infiltration and tumor microenvironment, especially in glioma. Moreover, si-RNA mediated knockdown of CHD5 promoted the proliferation and migration of glioma cells in vitro. In conclusion, CHD5 was found to be differentially expressed in the pan-cancer analysis and might play an important role in antitumor immunity. CHD5 is expected to be a potential tumor prognostic marker, especially in glioma.

## 1. Introduction

CHD5, a member of the chromatin remodeling family, consists of nine proteins (CHD1-9) defined by a dual chromatin domain and a SWI/SNF-like ATP-dependent helical motif. As its closest members CHD3 and CHD4, CHD5 have a tandem PHD structure and are involved in the regulation of chromatin structure and transcription. Previous studies have suggested that PHD might be a “reader for specific modified or unmodified histones” [1,2,3,4,5].

Previous experimental data reported that CHD5 was closely associated with various types of malignancy. Downregulation of CHD5 has been shown to stimulate DNA damage response and predict poor prognosis in pancreatic cancer patients [6]. CHD5 shows low expression levels and exerts inhibitory effects in gastric cancer and leukemia [7,8]. CHD5 inhibits the progression in NSCLC, and inhibits the migration and invasion in colorectal cancer cells in vitro [9,10]. Moreover, low expression of CHD5 is known to be associated with adverse clinical and pathological features such as shorter overall and disease-free survival [11,12]. Although CHD5 is currently recognized as a potential biomarker and key mediator in multiple human malignancies, the potential mechanism underlying its antitumor function related to tumor immunity remain unclear.

In this study, we analyzed the expression of CHD5 and its relationship with the prognosis, TMB and MSI in 33 cancer types. In addition, we examined the correlation between CHD5 and the immune microenvironment, immune-related antigens, and immune checkpoint genes. The results from our pan-cancer analysis revealed CHD5 was a tumor suppressor in multiple cancer types, and low expression of CHD5 may reduce the survival time of cancer patients. Furthermore, the knockdown of CHD5 promoted glioma cancer cell proliferation and migration in vitro. In conclusion, CHD5 is a promising therapeutic target in cancer and is a prognostic marker that is associated with immune infiltration in glioma.

## 2. Results

### 2.1. CHD5 Is Differentially Expressed between Tumor and Normal Tissues

**Figure 1:**
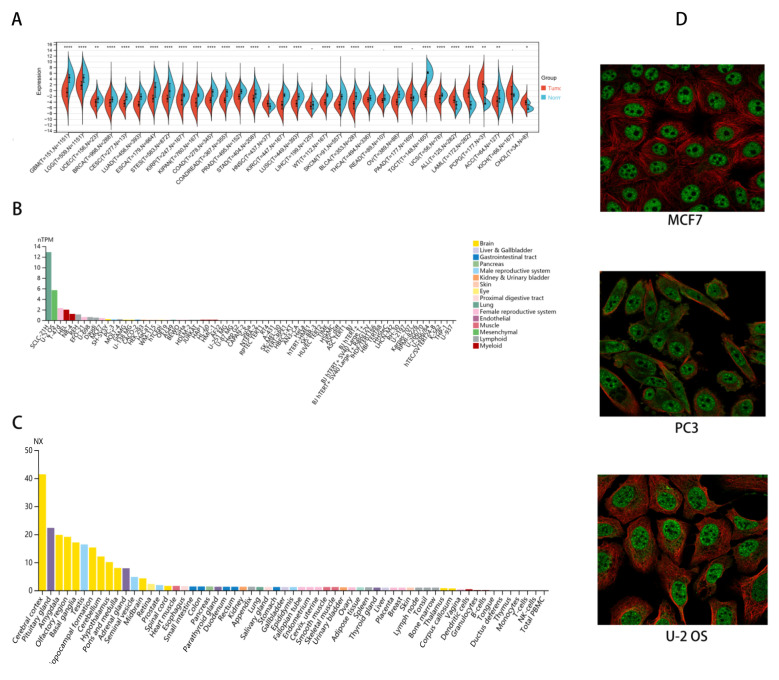
Pan-cancer analysis of *CHD5* expression. (**A**) Differential expression of *CHD5* between tumor and normal tissues in pan-cancer analysis. (**B**,**C**) Expression of *CHD5* in various cancer cell lines. (**D**) Cellular localization of CHD5 (*p* < 0.05). * *p* < 0.05; ** *p* < 0.01, **** *p* < 0.0001.

We obtained CHD5 expression data for 33 types of cancers. Differences in the expression of CHD5 between the normal and tumor samples in each tumor type were then calculated. We observed significant down regulation of CHD5 in 25 tumor types, including GBM, Glioma, LGG, UCEC, BRCA, CESC, LUAD, ESCA, STES, KIRP, KIPAN, COAD, COADREAD, PRAD, STAD, KIRC, LUSC, WT, SKCM, BLCA, THCA, OV, TGCT, UCS, and ACC, while there was a significant upregulation in 5 tumor types including HNSC, ALL, LAML, PCPG, and CHOL (*p* < 0.05) (Figure 1A). It was further found that CHD5 was downregulated in most cancer cells, with higher expression mainly in SCLC-21H (lung), U-2 OS (bone) and T-47d (breast) cancer cell lines (Figure 1B). Interestingly, histopathological studies showed that CHD5 was mainly expressed in the brain (Figure 1C), and it was mainly localized to the nucleoplasm and nuclear speckles within the cells (Figure 1D). Overall, our results showed that CHD5 expression was significantly downregulated in 25 types of cancers relative to the normal tissue and its expression levels were associated with neurological tumors.

### 2.2. Pan-Cancer Analysis of the Prognostic Value of CHD5

**Figure 2:**
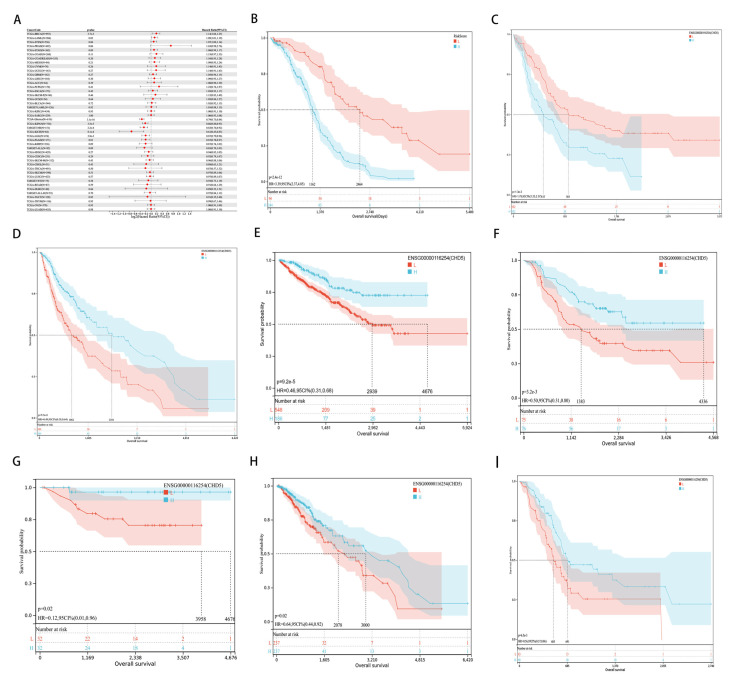
*CHD5* expression correlates with overall survival time (OS). (**A**) Forest plots showing the correlations between OS and *CHD5* expression across 44 types of cancers. Kaplan-Meier analyses of the association between *CHD5* expression and OS in (**B**) Breast invasive carcinoma (BRCA), (**C**) Acute Myeloid Leukemia (LAML), (**D**) Glioma, (**E**) Pan-kidney cohort (KICH+KIRC+KIRP) (KIPAN), (**F**) Neuroblastoma (NB), (**G**) Kidney Chromophobe (KICH), (**H**) Low Grade Glioma (LGG), and (**I**) Pancreatic adenocarcinoma (PAAD).

To further investigate the correlation between *CHD5* expression and prognosis, we performed a survival analysis for 33 types of cancers using the following metrics: overall survival (OS), disease-free survival (DSS), disease-free interval (DFI) and progression-free interval (PFI). Univariate Cox analysis showed that *CHD5* expression was associated with overall survival (*p* < 0.05) in BRCA, LAML, Glioma, KIPAN, NB, KICH, LGG, and PAAD (Figure 2A). Furthermore, Kaplan-Meier analysis indicated that the expression of *CHD5* was negatively correlated with the survival time in BRCA (Figure 2B, *p* < 0.01), and LAML (Figure 2C, *p* = 0.03). However, in Glioma (Figure 2D, *p* < 0.001), KIPAN (Figure 2E, *p* < 0.001), NB (Figure 2F, *p* < 0.001), KICH (Figure 2G, *p* < 0.001), LGG (Figure 2H, *p* < 0.05 (*p* = 0.02) and PAAD (Figure 2P, *p* <= 0.01) patients, the expression of *CHD5* was positively correlated with the survival time. In particular, when the optimal cut-off value was used for grouping, CHD5 could effectively distinguish the high-risk and low-risk groups in KIPAN.

**Figure A1:**
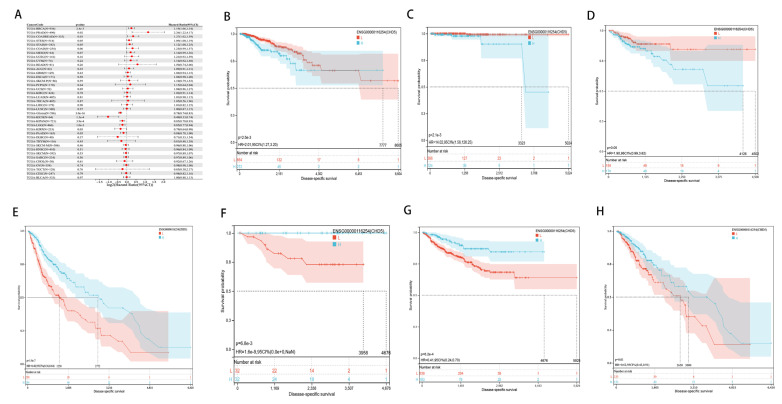
Correlation between *CHD5* expression and disease-specific survival (DSS). (**A**) Forest plots showing correlations between DSS and *CHD5* expression in 44 tumor types. Kaplan-Meier analyses of the association between *CHD5* expression and DSS in (**B**) Breast invasive carcinoma (BRCA), (**C**) prostate cancer (PRAD), (**D**) Colon adenocarcinoma/Rectum adenocarcinoma Esophageal carcinoma (COADREAD), (**E**) Glioma, (**F**) Kidney Chromophobe (KICH), (**G**) Pan-kidney cohort (KICH+KIRC+KIRP) (KIPAN), (**H**) low grade glioma (LGG).

**Figure A2:**
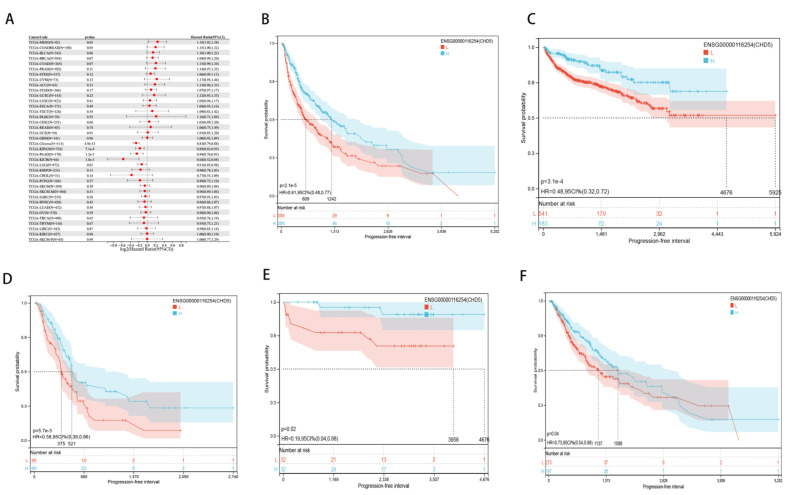
Correlation between *CHD5* expression and disease-free interval (DFI). (**A**) Forest plots showing the relationship between *CHD5* expression and DFI in 44 tumor types. Kaplan-Meier analyses of the association between *CHD5* expression and DFI in (**B**) Glioma, (**C**) Pan-kidney cohort (KICH+KIRC+KIRP) (KIPAN), (**D**) Pancreatic adenocarcinoma (PAAD), (**E**) Kidney Chromophobe (KICH), (**F**) low grade glioma (LGG).

In addition, DSS analysis showed that *CHD5* expression was associated with the prognosis of BRCA, PRAD, COADREAD, Glioma, KICH, KIPAN, LGG and KIRP (*p* < 0.05) (Figure A1A). Among these analyses, the KICH group showed the lowest hazard ratio (HR = 0.49). Kaplan-Meier analysis also showed that high *CHD5* expression was associated with poor prognosis in BRCA (Figure A1B, *p* < 0.01), PRAD (Figure A1C, *p* < 0.01) and COADREAD (Figure A1D, *p* = 0.05). However, the opposite association was observed in Glioma (Figure A1E, *p* < 0.001), KICH (Figure A1F, *p* < 0.01), KIPAN (Figure A1G, *p* < 0.001) and LGG (Figure A1H, *p*= 0.01) patients. Among the BRCA, KIRP, KIPAN and PRAD cancers, the optimal cut-off value had to be taken to obtain effective risk group differentiation. We further detected a correlation between high *CHD5* expression and lower DFI in MESO (*p* = 0.03) and COADREAD (*p* = 0.05), while in Glioma (*p* < 0.001), KIPAN (*p* < 0.001), PAAD (*p* < 0.01), KICH (*p* < 0.01) and LGG (*p* = 0.01), high *CHD5* expression was correlated with a higher DFI (Figure A2A). Kaplan-Meier analysis also showed that high *CHD5* expression was significantly associated with increased DFI in Glioma (Figure A2B, *p* < 0.001), KIPAN (Figure A2C, *p* < 0.001), PAAD (Figure A2D, *p* < 0.01), KICH (Figure A2E, *p* = 0.02) and LGG (Figure A2F, *p* = 0.04).

**Figure A3:**
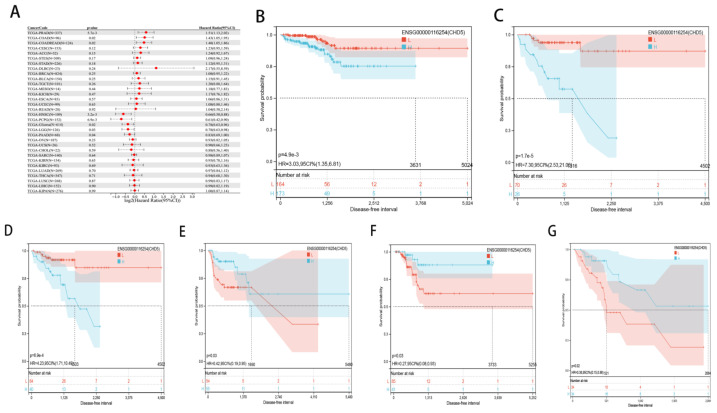
Correlation between *CHD5* expression and progression-free interval (PFI). (**A**) Forest plots showing the association between *CHD5* expression and PFI in 44 tumor types. Kaplan-Meier analyses of the association between *CHD5* expression and PFI in (**B**) Glioma, (**C**) Pan-kidney cohort (KICH+KIRC+KIRP) (KIPAN), (**D**) Pancreatic adenocarcinoma (PAAD), (**E**) Kidney Chromophobe (KICH), (**F**) low grade glioma (LGG), (**G**) Pancreatic adenocarcinoma (PAAD).

Forest plots indicated that the expression of *CHD5* was significantly correlated with PFI in PRAD (*p* < 0.001), COAD (*p* = 0.02), COADREAD (*p* = 0.02), HNSC (*p* < 0.01), PCPG (*p* < 0.01), Glioma (*p* = 0.02), LGG (*p* = 0.03), and PAAD (*p* < 0.04) (Figure A3A). KM survival analysis also revealed that *CHD5* expression was associated with reduced PFI in PRAD (Figure A3B, *p* < 0.01), COAD (Figure A3C, *p* < 0.001) and COADREAD (Figure A3D, *p* < 0.001) patients. Meanwhile, increased *CHD5* expression was associated with better PFI in individuals with HNSC (Figure A3E, *p* = 0.03), LGG (Figure A3F, *p* = 0.03) and PAAD (Figure A3G, *p* = 0.02). Finally, we summarized and intersected the results from the analysis of these four prognostic data, and found that *CHD5* showed strong prognostic correlation in CNS tumors, especially Glioma. Overall, we observed that *CHD5* expression might be strongly associated with the prognosis of patients with neurological tumors.

### 2.3. Correlations between CHD5 Expression and Pan-Cancer Clinicopathological Characteristics

**Figure 3:**
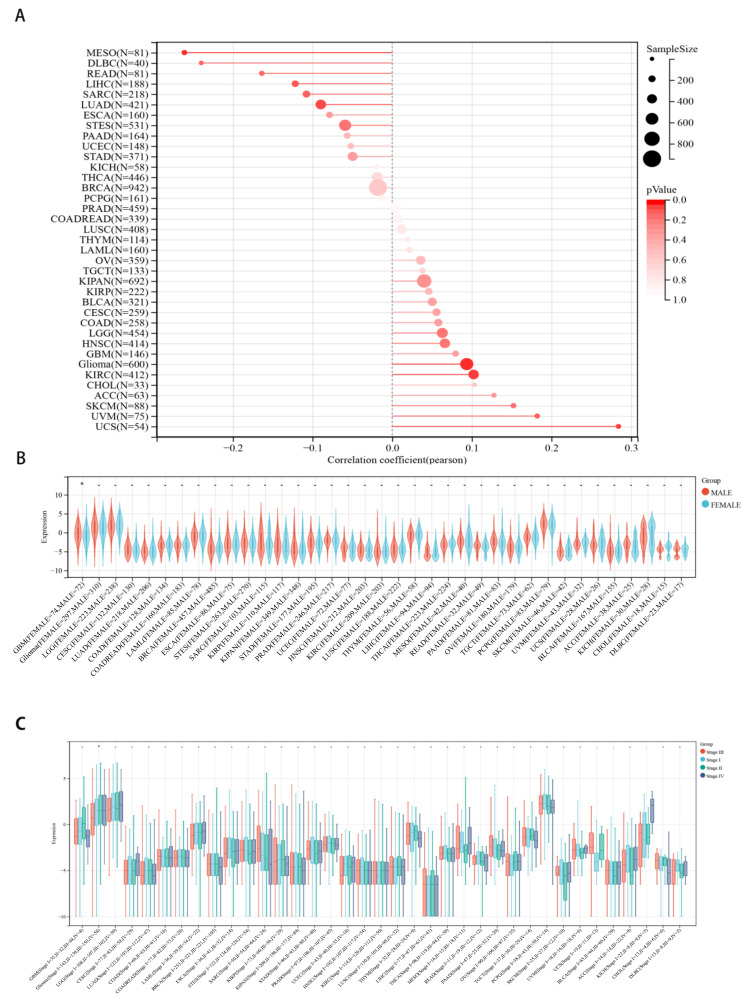
Correlations between *CHD5* expression and pan-cancer clinicopathology. (**A**) The expression of *CHD5* correlates with patient’s age in Glioma (*p* = 0.02), KIRC (*p* = 0.03), UCS (*p* = 0.03) and MESO (*p* = 0.01). (**B**) The expression of *CHD5* was correlated with gender (*p* < 0.05). (**C**) *CHD5* was significantly differentially expressed across different stages of Glioma (*p* = 0.04). * *p* < 0.05.

Next, we evaluated the differences in the expression of CHD5 in patients with different tumor types. The results showed that the expression of *CHD5* was significantly positively correlated with the age in three tumor types, namely, Glioma (*p* = 0.02), KIRC (*p* = 0.03), and UCS (*p* = 0.03), and was significantly negatively correlated in MESO (*p* = 0.01) (Figure 3A). In addition, we also observed that the expression of *CHD5* in GBM was sex dependent (*p* < 0.05) (Figure 3B), and significantly differentially expressed (*p* = 0.04) during different stages of Glioma (Figure 3C). No statistical differences were found in the other cancer types.

### 2.4. Correlation between CHD5 Expression and CNV, TMB, MSI in Various Cancer Types

**Figure 4:**
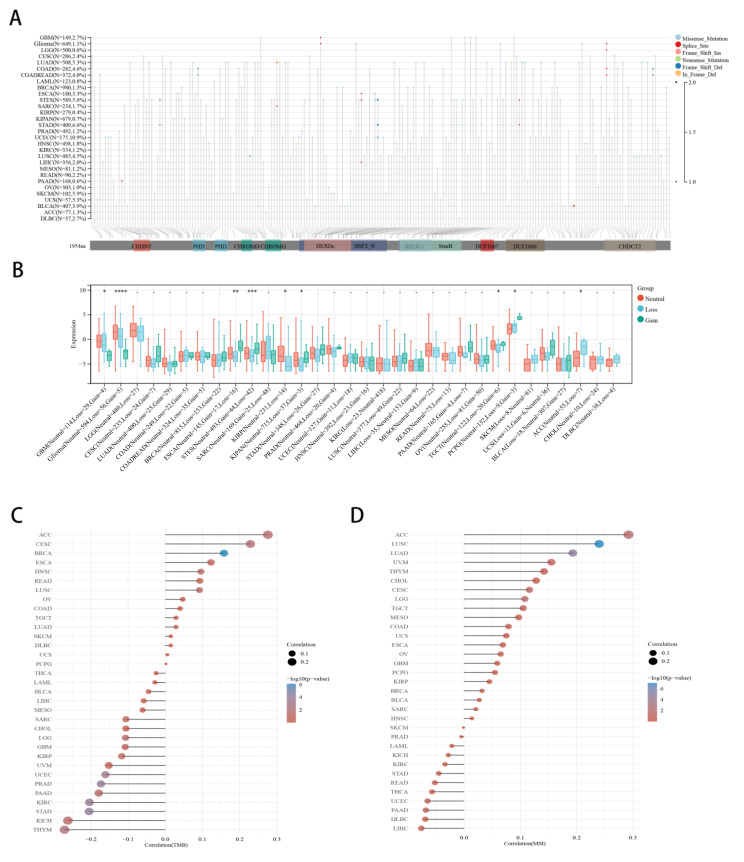
Correlation between *CHD5* expression and CNV, TMB, and MSI in various cancer types. (**A**) Landscape of *CHD5* mutation in 30 cancer types, (**B**) The CNV landscape of *CHD5* mutations in 32 types of cancers, (**C**,**D**) Spearman correlation analysis for TMB, MSI and *CHD5* gene expression. In the figure, the horizontal axis represents the correlation coefficient between the genes and TMB, and the vertical axis represents the different tumors. The size of the dots in the figure represents the correlation coefficient, and the different colors represent the significance of the *p* value. The bluer the color in the diagram, the smaller the *p* value. * *p* < 0.05, ** *p* < 0.01, *** *p* < 0.001, **** *p* < 0.0001.

We mapped the mutational profiles of *CHD5* across 30 types of cancers (Figure 4A). The results showed that UCEC (10.9%) cancer had the highest mutation rate, while LUAD (5.3%), COAD (4.6%), STES (5.6%), STAD (6.6%), SKCM (N = 102, 5.9%) and UCS (N = 57, 5.3%) had considerable mutations as well. We further explored whether CHD5 expression was dependent on the CNV status in the 32 types of cancers, and found statistically significant differences in 9 types of cancers (Figure 4B), including GBM (*p* = 0.02), Glioma (*p* = 5.1 × 10^−5^, ESCA (*p* = 4.6 × 10^−3^), STES (*p* = 5.6 × 10^−4^), KIRP (*p* = 0.02), KIPAN (*p* = 0.04), TGCT (*p* = 0.03), PCPG (*p* = 0.05) and ACC (*p* = 0.04). Notably, the *l*oss of *CHD5* occurred frequently in the genome, which affected the copy number of *CHD5* in glioma (Loss = 56, Gain = 5), and was one of the reasons for the low expression of *CHD5* in Glioma. This was also the case with GBM (Loss = 29, Gain = 4). Furthermore, the expression of *CHD5* was correlated with TMB in 12 types of cancers (Figure 4C). That is, it was positively correlated with the TMB for BRCA, CESC, ACC, and HNSC, and negatively correlated with STAD, PRAD, KIRC, UCEC, THYM, LGG, PAAD, and KICH. We further found that *CHD5* expression was positively correlated with MSI in 5 types of cancers, including LUSC, LUAD, ACC, LGG, and CESC (Figure 4D), but not negatively correlated with MSI in other types of cancers. These results also suggested that *CHD5* expression was closely associated with Glioma. 

### 2.5. Correlation between CHD5 Expression and the TME in Different Types of Cancers

**Figure 5:**
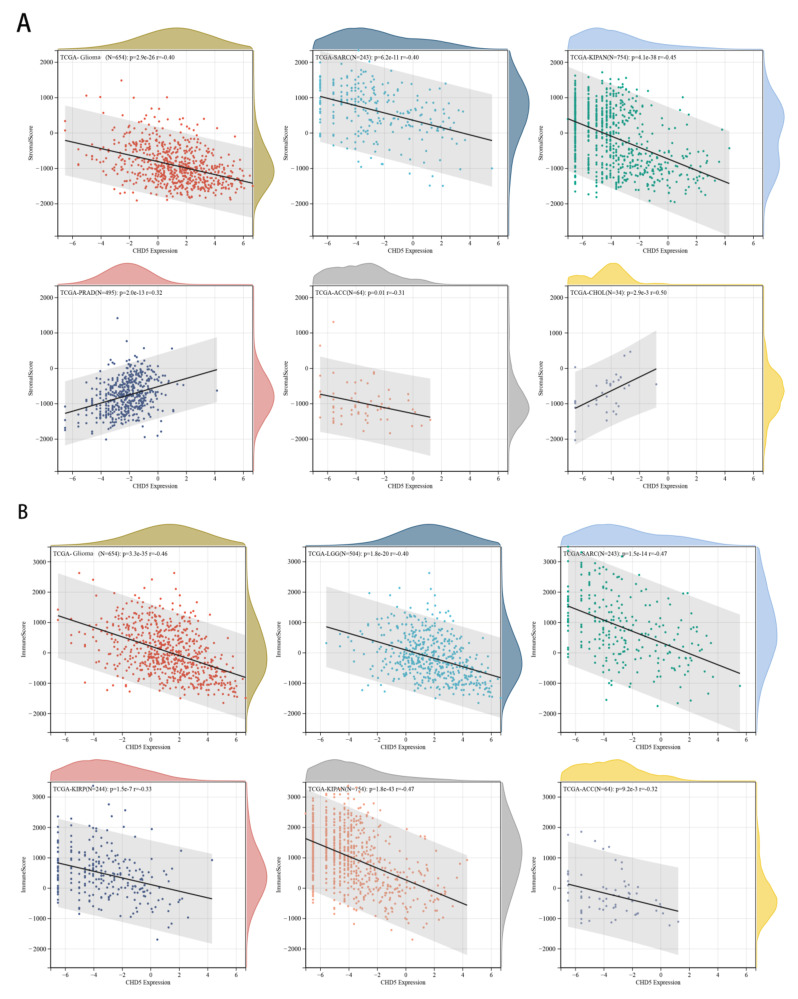
Relationship between *CHD5* expression and tumor microenvironment factors. (**A**) *CHD5* expression was negatively correlated with the stroma scores in Glioma, SARC, KIPAN, and ACC, and was positively correlated with the stroma scores in PRAD and CHOL. (**B**) *CHD5* expression was negatively correlated with the immune scores in Glioma, LGG, SARC, KIRP, KIPAN and ACC.

**Figure A4:**
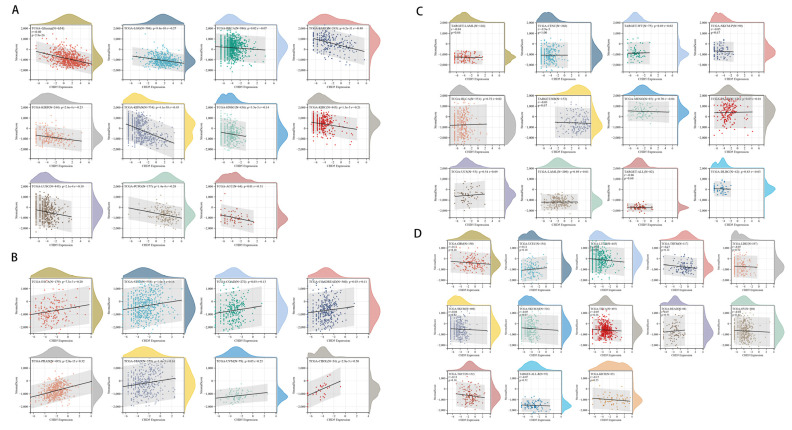
Correlation between *CHD5* expression and pan-cancer stromal score. (**A**) *CHD5* expression was negatively correlated with stroma scores for KIPAN, SARC, Glioma, ACC, PCPG, LGG, KIRP, KIRC, LUSC, HNSC, and BRCA. (**B**) The expression of *CHD5* was significantly positively correlated with the stromal scores of COADREAD, STES, STAD, ESCA, UVM, PRAD and CHOL. (**C**,**D**) No significant differences were found in other cancers.

**Figure A5:**
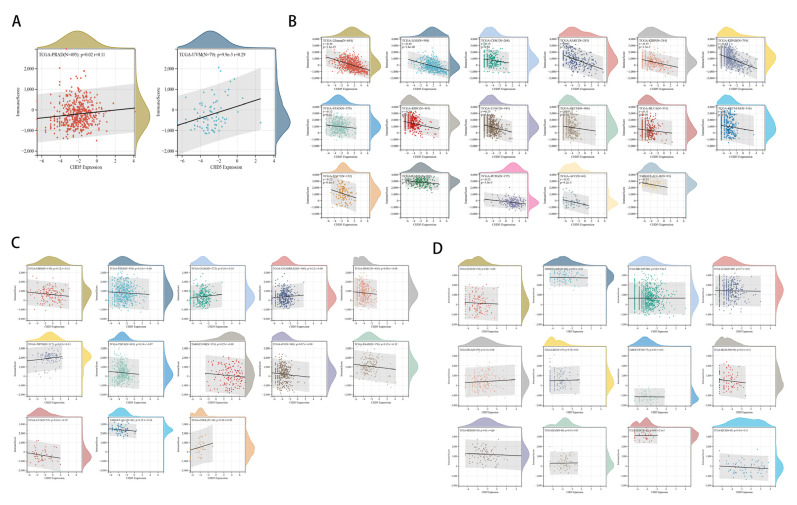
Correlation between *CHD5* expression and pan-cancer immune score. (**A**) *CHD5* expression was negatively correlated with immune scores in KIPAN, SARC, Glioma, LGG, KIRP, ACC, KIRC, LUSC, ALL, TGCG, PCPG, LAML, CESC, STAD, SKCM-M, BLAC and SKCM, (**B**) *CHD5* expression was significantly positively correlated with the immune scores in PRAD and UVM. (**C**,**D**) No significant differences were found in other cancers.

The role of the tumor microenvironment (TME) in the occurrence and development of tumors has been demonstrated before [13,14]. Genetic alterations in the cancer cells are the primary cause for their uncontrolled growth, resistance to apoptosis, and metabolic shift to anaerobic glycolysis (Warburg effect). These events trigger a series of metabolic, and immune events, which ultimately cause cancer progression and metastasis [15]. Therefore, in our pan-cancer analysis, we evaluated the association between CHD5 expression and the TME. The stroma score is a method to evaluate the content of stromal cells in the tumor tissue, and the content of stromal cells often reflects the malignancy of the tumor [16]. Our results showed that *CHD5* expression was negatively correlated with the stroma score in KIPAN, SARC, Glioma, ACC, PCPG, LGG, KIRP, KIRC, LUSC, HNSC, and BRCA (Figure A4A), and it was significantly positively correlated with the stroma score in COADREAD, STES, STAD, ESCA, UVM, PRAD and CHOL (Figure A4B). No significant differences were found in other cancers (Figure A4C,D). The tumor microenvironment is composed of fibroblasts, endothelial cells, tumor cells, immune cells, and vasculature, which play important roles in tumor progression. Immune score is a method to evaluate the tumor immune microenvironment, which has been demonstrated to be superior to the AJCC/UICC TNM-classification in colorectal cancer [17,18]. Furthermore, CHD5 expression found to be negatively associated with the immune scores in KIPAN, SARC, Glioma, LGG, KIRP, ACC, KIRC, LUSC, ALL, TGCG, PCPG, LAML, CESC, STAD, SKCM-M, BLAC and SKCM (Figure A5A). There was a significant positive correlation between *CHD5* expression and the immune score in PRAD and UVM (Figure A5B). No significant differences were found in other cancers (Figure A5C,D). The 6 types of cancers with the highest correlation coefficient between the TME and *CHD5* expression are shown in Figure 5A,B.

### 2.6. The Relationship between CHD5 Expression and Immune Cell Infiltration in Various Cancer Types

**Figure 6:**
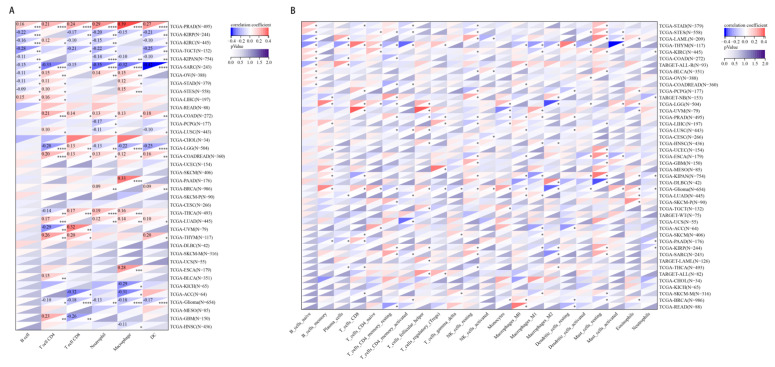
Pan-cancer analysis of the relationship between *CHD5* expression and immune cell infiltration. (**A**) Correlation between *CHD5* expression and B cell, CD4+T cell, CD8+ T cell, neutrophil, macrophage, DC infiltration in each patient. (**B**) *CHD5* expression was significantly associated with immune cell infiltration in 41 types of cancers. * *p* < 0.05; ** *p* < 0.01, *** *p* < 0.001, **** *p* < 0.0001.

Finally, we obtained six immune cell infiltration types from 8590 tumor samples across 38 tumor types, and showed that *CHD5* gene expression was significantly correlated with immune infiltration in 28 types of cancers (Figure 6A). Notably, *CHD5* expression was significantly positively correlated with the six immune cell infiltration types in PRAD and was negatively correlated with the same in SARC. Consistent with previous studies, the expression of *CHD5* gene in Glioma was significantly negatively correlated with the 5 immune cells types, except B cells. Next, we examined the relationship between *CHD5* expression and the infiltration of 22 immune cell subtypes. The findings showed that in most cancer types (41/44), the level of immune cell infiltration was significantly positively correlated with the expression of CHD5 (Figure 6B). In Glioma, up to 16 types of immune cells were associated with *CHD5* expression. The above results implied that there was an association between *CHD5* expression and the degree of immune cell infiltration in different types of tumors.

### 2.7. Knockdown of CHD5 Promotes the Proliferation, Migration and EMT in GBM Cells In Vitro

**Figure 7:**
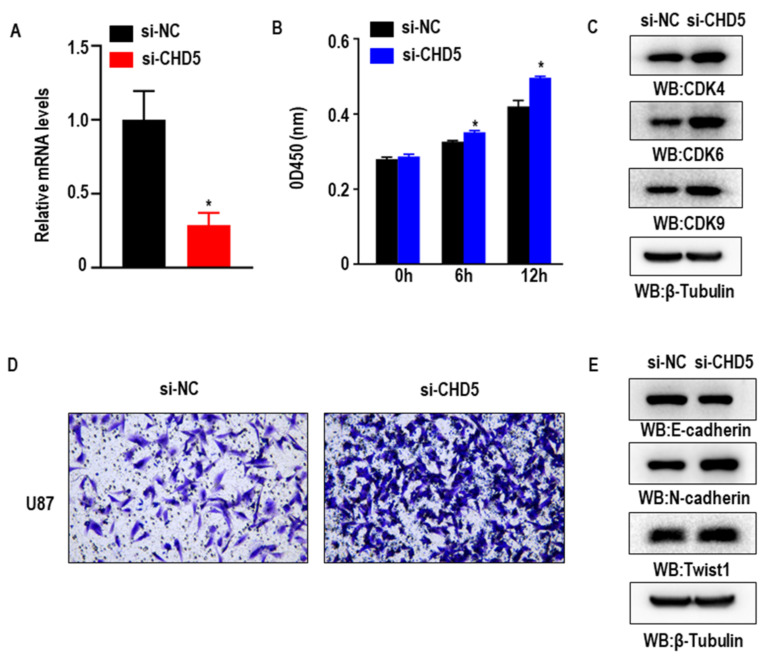
Cellular functions of CHD5. (**A**) Real-time quantitative PCR detection after siRNA mediated knockdown of *CHD5* in U87 cells. (**B**,**C**) CCK-8 assay results showing the increase in the viability of U87 cells upon the knockdown of *CHD5*. (**D**,**E**) Transwell assay results showing the increase in the migration and EMT progression in U87 cells upon the knockdown of *CHD5*. * *p* < 0.05.

To investigate the biological function of CHD5 in glioblastoma progression, we subsequently performed loss-of-function experiments and silenced the expression of *CHD5* in the human GBM cell line U87. RT-qPCR confirmed the efficiency of siRNA knockdown of *CHD5* (Figure 7A). CCK8 assay for cell viability showed that the knockdown of *CHD5* increased the viability of U87 cells in vitro (Figure 7B). Consistent with this, the expression of cell cycle-related proteins CDK4, CDK6, and CDK9 were also found to be increased (Figure 7C). Moreover, results from the transwell migration assay also showed that the knockdown of *CHD5* significantly promoted the migration and EMT process in GBM cells (Figure 7D,E).

## 3. Discussion

CHD5 has previously been reported to be a potential tumor suppressor gene in various solid tumors [19]. Nonetheless, few systematic studies have evaluated the roles of CHD5 through pan-cancer analysis using bioinformatics approaches. This study aimed to systematically determine the expression pattern, prognostic value and potential function of CHD5 in different types of cancers.

In the present study, we first demonstrated that *CHD5* was differentially expressed between most cancer tissues and the adjacent normal tissues, which suggested that *CHD5* was a potential tumor suppressor gene in most cancers. The above finding was consistent with previously reported results regarding the differential expression of *CHD5* in different cancers, including neuroblastoma, melanoma, hepatocellular carcinoma, non-small cell lung cancer, and head and neck squamous cell carcinoma [20,21,22,23,24]. Our results also showed that the expression of *CHD5* was upregulated in some cancer types. Given the expression of *CHD5* was restricted to the transcriptional level, and its function at the protein level might have a major impact on cancer progression, it is critical to further explore the effect of the protein expression of CHD5 across different types of cancers. In other studies, CHD5 was reported to be more preferentially expressed in tumors in the nervous system [25], especially in neuroblastoma with a deletion of the 1p36.3 region [26], and was shown to be a potential tumor suppressor gene in neuroblastoma [27]. However, the role of CHD5 in glioma remains unclear. Therefore, it is necessary to further explore the function of CHD5 in apoptosis, proliferation and tumor migration using various cell biology experimental approaches in the future.

In the pan-cancer survival analysis, although CHD5 expression was associated with the prognosis of various cancers, it appeared to be strongly associated with the prognosis of neurological tumors (Glioma, LGG, and NB). However, several of the previous studies have demonstrated that CHD5 was a tumor suppressor gene in neuroblastoma, and was associated with a poor prognosis [27,28,29]. Interestingly, in this study, we revealed the biological role of CHD5 in glioma cells, wherein we found that the knockdown of *CHD5* enhanced the proliferation and migration of glioma cells in vitro.

To further explore the mutational landscape of CHD5 in different cancers, we analyzed the data from 30 cancers in the TCGA database. The results showed that the highest mutation rate was observed in UCEC (10.9%), and there was also a high mutation rate in GBM (*p* = 0.02), Glioma (*p* = 5.1 × 10^−5^), ESCA (*p* = 4.6 × 10^−3^), STES (*p* = 5.6 × 10^−4^), KIRP (*p* = 0.02), KIPAN (*p* = 0.04), TGCT (*p* = 0.03), PCPG (*p* = 0.05), and ACC (*p* = 0.04). TMB is a potential biomarker for multiple cancers, which is measured by the total amount of somatically encoded mutations [30,31]. Previous evidence has shown that tumors with high TMB were sensitive to immunotherapy and were associated with improved survival [32,33]. In this study, we found a positive correlation between CHD5 expression and TMB of BRCA, CESC, ACC, and HNSC, and a negative correlation with that of TMB in STAD, PRAD, KIRC, UCEC, THYM, LGG, PAAD, and KICH. MSI, defined as a phenotype of altered microsatellite sequences caused by defects in DNA mismatch repair, is associated with increased susceptibility to cancer [34]. In recent years, MSI has been recognized as the primary biomarker for immune checkpoint blockade therapy [35]. Our findings showed that *CHD5* expression was positively correlated with MSI in 5 types of cancers, including LUSC, LUAD, ACC, LGG, and CESC, but was not negatively correlated with any of the cancer types. Currently, the CNV, TMB and MSI of CHD5 are less reported in these cancers, and our study provides new insights into the mutational landscape in these cancers. Additionally, CHD5 was found to be strongly associated with tumors of Glioma. Notably, we found that the expression of *CHD5* was positively correlated with the MSI in LUSC, LUAD, ACC, LGG, and CESC, however, the MSI rate in LUAD and LGG was not high. At the same time, the expression of *CHD5* was not found to correlate with their TMB. CHD5, which has been speculated in past studies to function as a NuRD-type chromatin remodeling complex (transcriptional repressor), may also contribute to transcriptional activation, as well as transcriptional elongation, termination, RNA processing and DNA damage response [19,36]. Whether this suggests that CHD5 dominates DNA damage repair in LUAD and LGG leading to MSI is worthy of further investigation.

Tumorigenesis is highly correlated with the tumor microenvironment (TME), and in recent years strategies to therapeutically target the TME have emerged as a promising cancer therapy [37]. Glioma tumor immune microenvironment plays an important role in the progression of glioma. Our results showed that *CHD5* expression was positively correlated with the stromal scores in 18 types of cancers, was correlated with the immune scores in 19 types of cancers, and was especially significantly negatively correlated with the immune scores in Glioma. Moreover, we observed that *CHD5* expression was significantly associated with immune infiltration in 28 types of cancers. Notably, *CHD5* expression was significantly positively correlated with six immune cell infiltration types in PRAD and was negatively correlated in SARC. Recent studies have shown that B cells were associated with patient survival in sarcoma and their response to immunotherapy [38,39]. The expression of *CHD5* in Glioma was significantly negatively correlated with 5 types of immune cells, except B cells. However, myeloid-derived suppressor cells promote B cell-mediated immunosuppression through PD-L1 transfer in glioblastoma [40], which indicated that the tumor suppressor effect of CHD5 may not be through B cell immune function. Next, we examined the relationship between *CHD5* expression and the infiltration of 22 immune cell subtypes. The findings showed that in most cancer types, the level of immune cell infiltration was significantly correlated with the expression of *CHD5*. To our surprise, as many as 16 immune cell subtypes were associated with the expression of the *CHD5* in Glioma. This was not only consistent with our previous results, but also suggested that the way *CHD5* functioned in tumors might be related to other non-B cell immune pathways. Moreover, as glioma-associated microglia and macrophages (GAMs) are known to be the major components in the TME, a switch from a pro-tumorigenic M2 phenotype to tumoricidal M1 phenotype may offer an opportunity to improve the efficacy of existing cancer therapy. Previous studies have reported the presence of many cellular factors, which were potentially involved in the cross-talk between GBM cells and the microglia including chemokines, cytokines, and miRNAs [41]. However, the mechanisms underlying the crosstalk between CHD5 and the microglia need to be explored at greater depth in future studies. Our first pan-cancer analysis of *CHD5* revealed that the gene was lowly expressed in most tumor tissues compared with adjacent normal tissues and that there was an association between *CHD5* expression and clinical prognosis. Our findings also suggested that *CHD5* might be an independent prognostic factor in multiple cancers and that the low expression of *CHD5* was associated with poor prognosis in major tumor types. Although bioinformatics analysis provided us with some important insights about the role of *CHD5* in malignancies, we further validated the tumor suppressor role of *CHD5* in glioma using molecular biology methods. Further in vitro and in vivo experiments are required to confirm our findings. Although we analyzed and integrated information from multiple databases, there were some limitations to our study. For example, the TCGA database mainly included the data from Caucasian patients, while data from patients belonging to other ethnicities was relatively scarce. In conclusion, the present study demonstrates that *CHD5* expression affects glioma progression and migration. Therefore, *CHD5* may be a promising biomarker for glioma to predict patient prognosis and efficacy of anticancer therapy. These findings may lead to personalized treatments for glioma patients and for other cancer patients harboring *CHD5* variants.

## 4. Materials and Methods 

### 4.1. Analysis of the Expression Level of CHD5 in the Pan-Cancer Datasets

We downloaded the unified and standardized pan-cancer datasets from the UCSC (https://xenabrowser.net/) database (accessed on 23 September 2021), including the TCGA, TARGET and GTEx databases [42,43,44]. A total of 19,131 samples and 60,499 gene expression data were included. Expression data of CHD5 (ENSG00000116254) gene in each sample was extracted. Subsequently, we utilized the Can SAR BLACK Tool (https://cansarblack.icr.ac.uk/ Version: 1.5.0; accessed on 23 September 2021) to study CHD5 mRNA expression in different cancer cell lines from the Cancer Cell Lines Encyclopedia (CCLE, https://portals.broadinstitute.org/ccle; accessed on 23 September 2021) Expression profile [45]. The Human Protein Atlas (HPA) was used to study the expression and cellular localization of CHD5 protein in different organs. The authors state clearly that we are unable to classify the cases in line with the 2021 WHO classification.

### 4.2. Identification of the Correlation between CHD5 Expression Levels and Clinicopathological Characteristics and Survival in Human Cancers

We extracted several metrics (overall survival [OS], disease-specific survival [DSS], disease-free [DFI], and progression-free [PFI]) from the TCGA, TARGET, and GTEx samples to investigate the association between CHD5 expression and patient outcomes. We also excluded samples with an expression level of 0 and a follow-up period of less than 30 days. Survival analysis was performed using the Kaplan-Meier method and ROC curves (*p* < 0.05), and then the R packages “survival” was used to draw the survival curves. The R packages “survival” and “forestplot” were used for Cox analysis to determine the correlation between CHD5 expression and survival. The R packages “ggpubr” and “limma” were used for analyzing the correlation between CHD5 expression and the clinicopathological characteristics.

### 4.3. Association between CHD5 Expression and Tumor Mutational Burden (TMB) or Microsatellite Instability (MSI) across Different Types of Cancers

We downloaded simple nucleotide variation and copy number variation data from GDC (https://portal.gdc.cancer.gov/) (accessed on 23 September 2021), and the data were then processed using the MuTect2 software and the GISTIC software [46,47]. The domain information of the protein was obtained using the R software package maftools (version version 2.2.10). The Simple Nucleotide Variation dataset was used to map the mutational landscape of CHD5 in 30 tumor types, the Copy Number Variation dataset was used to analyze the relationship between CHD5 expression and CNV in 32 types of cancers. TMB were obtained from Vesteinn Thorsson et al. and MSI values were obtained from Russell Bonneville et al. [48,49].

### 4.4. Correlation between CHD5 Expression and the TME across Different Types of Cancers

We downloaded and analyzed the immune cell infiltration score from the TCGA Im muCellAI Database (http://bioinfo.life.hust.edu.cn/web/ImmuCellAI/) (accessed on 23 September 2021), and the TIMER2 database (http://timer.cistrome.org/) (accessed on 23 September 2021) [50]. Patients within each tumor type in the TCGA datasets were divided into two groups based on their median CHD5 expression levels to compare the extent of immune cell infiltration [51]. Finally, we analyzed the tumor purity and stromal/immune cell infiltration and classified them into tumor tissues of various tumor types (n = 44) based on CHD5 expression data using CIBERSORT, which was used to estimate the abundance of specific cells in hybrid cell populations using gene expression dataset [52].

### 4.5. Cell Culture and CHD5 Targeted siRNA Transfection 

The U87 (human glioma cell line) cells were cultured in RPMI 1640 medium (Gibco, Carls bad, CA, USA) supplemented with 10% fetal bovine serum (Biological Industries, Kibbutz Beit Haemek, Israel) and 1% penicillin/streptomycin (Invitrogen, Grand Island, NY, USA). The cells were cultured at 37 °C in a 5% CO2 incubator. The si-NC and si-CHD5 small interfering RNA (siRNA) were transfected into U87 cells with Lipofectamine 2000 transfection reagent (Invitrogen, Carlsbad, CA, USA), respectively. Briefly, U87 cells were randomly divided into two groups: the si-NC group transfected with the scrambled siRNA was considered as the negative control, the si-CHD5 group was transfected with the siRNA specific for CHD5 (target sequence AGAAGGTATTCCGTATGAA) (RiboBio, Guangzhou, China). Transfection experiments were performed when cells were 50% confluent in 6 well plates. Once the cells reached the desired confluence, the culture medium was aspirated and 1.5 mL complete medium was added to each well again. siRNA(30 nM) and lipo2000(5 μL) were added to 0.5 mL of serum-free medium, mixed and incubated at room temperature for 30 min. The siRNA-lipo2000 mixture was carefully added to each well. Then the cells were cultured at 37 °C in a humidified atmosphere with 5% CO2.

### 4.6. RNA Isolation and qPCR

After the cells were transfected for 48 h, the mRNA levels of CHD5 were analyzed by qPCR assay. Briefly, total RNA was extracted from the cells using the total RNA extraction kit (Bioflux, Tokyo, Japan). 1.5 µg of RNA was reverse transcribed into cDNA and the mRNA level was analyzed by UltraSYBR Master (CWBIO, Guangzhou, China). The qPCR primers for CHD5 were as follows: F-CCCCATGTCCAAAATGATGACC, and R-GTGACCGTCTCTACAGCCG. The data were normalized to 18 s and the relative gene expression was obtained using Prism 7 (GraphPad Software, lnc., La Jolla, CA, USA).

### 4.7. Cell Viability Assay

After the cells were transfected for 48 h, the effect of CHD5 knockdown on the viability of U87 cells was assessed using the cell counting kit-8 (CCK-8) assay (MedChem Express, Shanghai, China). In brief, the cells were seeded in 96-well plates at a density of 2 × 10^4^ cells per well and cultured for 0 h, 6 h, 12 h. Following this, RPMI 1640 medium (100 μL) and CCK8 solution (10 μL) were added to each well, and incubated for 1 h. Finally, the absorbance of each well was measured at 450 nm using a microplate reader (Thermo Scientific, Waltham, MA, USA). The data was analyzed using the ImageJ2x Software (Rawak Software Inc., Stuttgart, Germany).

### 4.8. Transwell Assay

To evaluate the effect of CHD5 knockdown on U87 cells migration, U87 cells were transfected with si-NC and si-CHD5 siRNAs, respectively, according to the manufacturer’s instructions. After the cells were transfected for 48 h, a total of 1 × 10^4^ cells were diluted in RPMI 1640 medium without FBS and were plated into the transwell chamber (Corning, Kennebunk, ME, USA). The chambers were placed in 24 well plates containing 600 μL complete medium. After 24 h, the cells that migrated to the bottom of the membrane were fixed with 4% paraformaldehyde for 30 min. Then, the membranes were stained with 0.1% crystal violet (Sangon Biotech, Shanghai, China). Eight fields were captured randomly under a microscope (DMI8, Leica, Wetzlar, Germany).

### 4.9. Western Blot Analysis

The si-NC and si-CHD5 siRNA were transfected into U87 cells with Lipofectamine 2000 reagent. After the cells were transfected for 48 h, Western blot analysis was performed as previously described [53]. Briefly, the cells were lysed with 1% SDS lysing buffer containing protease inhibitor cocktail and phosphatase inhibitor cocktail (Apexbio, Houston, TX, USA). The protein concentration was determined using the BCA protein assay reagent kit (Thermo Scientific, Waltham, MA, USA). All the blots were incubated with the respective primary antibodies, namely, anti-β-Tubulin (1:5000, TRANSGEN, Beijing, China), anti-CDK4, anti-CDK6, anti-CDK9 (1:800, Cell Signaling Technology, Boston, MA, USA), anti-E-cadherin, anti-N-cadherin, and anti-Twist1 (1:1000, Bioworld, Nanjing, China) antibodies. The protein bands were visualized with ECL Reagents (Smart-Lifesciences, Nanjing, China).

## 5. Conclusions

In summary, our pan-cancer analysis revealed that *CHD5* was abnormally expressed in cancer samples across different types of cancers, and the abnormal expression of *CHD5* correlated with the clinicopathological features and patient prognosis, especially in tumors related to the nervous system. In addition, the TME, TMB, MSI, and immune infiltration might contribute to the dysregulation of *CHD5* expression in cancer, and CHD5 may be a potential therapeutic target for glioma immunotherapy.

## Data Availability

Data used in this study can be downloaded from TCGA (https://tcga-data.nci.nih.gov/tcga/), Ucsc Xena (https://xenabrowser.net/datapages/), CellMiner (https://discover.nci.nih.gov/cellminer/home.do), and Harmonizome (https://maayanlab.cloud/Harmonizome/) (accessed on 23 September 2021).

## Abbreviations

ACC	Adrenocortical carcinoma
BLCA	Bladder urothelial carcinoma
BRCA	Breast invasive carcinoma
CESC	Cervical squamous cell carcinoma and endocervical adenocarcinoma
CHOL	Cholangiocarcinoma
COAD	Colon adenocarcinoma
COADREAD	Colon adenocarcinoma/Rectum adenocarcinoma esophageal carcinoma
DLBC	Lymphoid neoplasm diffuse large B-cell lymphoma
ESCA	Esophageal carcinoma
FPPP	FFPE pilot phase II
GBM	Glioblastoma
HNSC	Head and neck squamous cell carcinoma
KICH	Kidney chromophobe
KIPAN	Pan-kidney cohort (KICH+KIRC+KIRP)
KIRC	Kidney renal clear cell carcinoma
KIRP	Kidney renal papillary cell carcinoma
LAML	Acute myeloid leukemia
LGG	Low grade glioma
LIHC	Liver hepatocellular carcinoma
LUAD	Lung adenocarcinoma
LUSC	Lung squamous cell carcinoma
MESO	Mesothelioma
OV	Ovarian serous cystadenocarcinoma
PAAD	Pancreatic adenocarcinoma
PCPG	Pheochromocytoma and paraganglioma
PRAD	Prostate adenocarcinoma
READ	Rectum adenocarcinoma
SARC	Sarcoma
STAD	Stomach adenocarcinoma
SKCM	Skin cutaneous melanoma
STES	Stomach and esophageal carcinoma
TGCT	Testicular germ cell tumors
THCA	Thyroid carcinoma
THYM	Thymoma
UCEC	Uterine corpus endometrial carcinoma
UCS	Uterine carcinosarcoma
UVM	Uveal melanoma
OS	Osteosarcoma
ALL	Acute lymphoblastic leukemia
NB	Neuroblastoma
WT	High-risk wilms tumor
TCGA	The Cancer Genome Atlas
GEO	Gene Expression Omnibus
OS	Overall survival
DFI	Disease free interval
DFS	Disease free survival
